# Zn(II) coordination polymers: therapeutic activity and nursing application values against coronary heart disease

**DOI:** 10.1080/15685551.2022.2086397

**Published:** 2022-06-09

**Authors:** Sheng-Min Sang, Min-Xia Zhang

**Affiliations:** a Department of Cardiology, Affiliated Hai’an Hospital of Nantong University (Haian People’s Hospital), Hai’an, Jiangsu, China; b Department of Nursing, Shangluo Traditional Chinese Medicine Hospital, Shangluo, Shaanxi, China

**Keywords:** Coordination complex, coronary heart disease, arterial endothelial cells

## Abstract

In the current research, two coordination polymers (CPs) have been produced solvothermally on the basis of a semi-rigid multifunctional tricarboxylate, i.e., 5-(3,4-dicarboxylphenoxy) nicotic acid (H_3_L), and the chemical compositions of the two compounds are [Zn(H_2_L)_2_(H_2_O)_2_] **1** and [Zn(HL)(2,2'-bpy)] (**2**, 2,2'-bpy = 2,2'-bipyridine), respectively. The structures and CHN analysis of both complexes were researched. The structural analysis results show that complex **1** features a 2D layered network with **sql**-type topology and complex **2** demonstrates a 2D layered network with uninodal **hcb** topology. The therapeutic activity and nursing application values of compounds against coronary heart disease were explored, and their relevant mechanism was assessed in meantime. The endothelin (ET) and prostacyclin (PGI2) contents released by the arterial endothelial cells into plasma were determined with ELISA assay. In addition to this, the alpha granule membrane protein 140 (GMP140) on the platelet was determined with real-time RT-PCR assay.

## Introduction

The occurrence of coronary heart disease is a complex inflammatory process under the action of multiple factors. Coronary heart disease (CHD) is a common disease of the cardiovascular system, and its pathophysiological basis is atherosclerosis (AS) [1]. Due to the in-depth understanding of the biological functions of vascular endothelial cells (VEC) in recent years, the focus of research on the pathogenesis of AS and CHD has gradually shifted to VEC dysfunction [2].

**Scheme 1.**
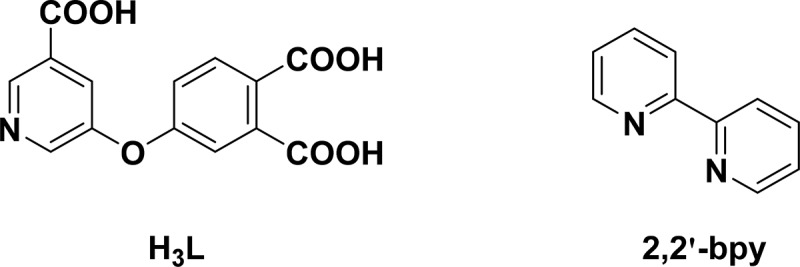
The chemical drawings for the organic ligands in this work.

Coordination polymers (CPs) composed of organic connectors and metal cations have many advantages, for instance, adjustability, porosity and the diversity in structure. They are a kind of classical materials with extensive application prospects. As a result, it is a significant goal for the chemists to generate CPs having latent application prospects and specific architecture [3–5]. It is well known that the generation of the CPs is affected through various factors, for example, metal ions, solvents, organic ligands, the ratio of counterions and reactants, etc. The choice of the organic ligands is a crucial factor, on account of their steric hindrance effects, length, flexibility and rigidity, as well as the shape will influence the performances and architecture of the final skeletons directly [6–8]. Up to now, the aromatic polycarboxylic acid ligands have been confirmed as the most notable connectors for the establishment of fresh CPs owing to their multifunctional conformation of coordination and strong ability of coordination with a variety of the metal ions [9–12]. The planar or tetrahedral tetracarboxylic acids, trigonal tricarboxylic acids, linear dicarboxylic acids, octacarboxylic acids, together with hexacarboxylic acids and other polycarboxylic acid connectors, have been applied for the establishment of fresh CPs. In recent years, a type of semi-rigid carboxylate molecules with V shape have received much attention [13–15]. The semi-rigid carboxylic acids with V shape containing extra *O*-ether functional groups and/or *N*-pyridyl among these polycarboxylic acid blocks are especially interesting on account of their probable result in CP creation with unusual architectures [16–20]. On the other hand, as a borderline Lewis acid, zinc is a component of many metalloenzymes and has a specific role in the bioinorganic processes of these enzymes. Unlike iron and copper that have different oxidation states, Zn(II) ion plays only a structural role in forming appropriate enzymes by means of coordination to amino acids such as cysteine and histidine in an approximately tetrahedral coordination geometry. Its d^10^ configuration and zero ligand field stabilization energy give it a potential to adopt four, five, or sixcoordination, without a marked preference for six coordination when a zinc enzyme interacts with a substrate. In order to design and synthesize model compounds of the zinc enzymes, many coordination chemistry researchers have focused their work on the synthesis of Zn (II) complexes with nitrogen, oxygen and sulfur donor ligands, which can mimic the active site of these enzymes. Zinc is the only metal ion that can facilitate the rewinding of DNA. Additionally, many zinc complexes have shown anticonvulsant, antidiabetic, anti-inflammatory, antimicrobial, antioxidant and anticancer properties, and some of them have also been tested for the treatment of Alzheimer’s disease. Based on the above considerations, in the current research, two coordination polymers (CPs) [Zn(H_2_L)_2_(H_2_O)_2_] **1** and [Zn(HL)(2,2'-bpy)] (**2**, 2,2'-bpy = 2,2'-bipyridine, Scheme 1) have been produced solvothermally on the basis of the semi-rigid multifunctional tricarboxylate, i.e., 5-(3,4-dicarboxylphenoxy) nicotic acid (H_3_L). The structures and CHN analysis of both complexes were researched. After serial biological experiments, the created compounds’ therapeutic activity and nursing application values were explored and their mechanism was also investigated.

## Experimental

### Chemicals and measurements

The ligands used in this study are supplied by Shanghai Chemsoon Chemical Reagent Company, and all other starting chemicals employed in our investigation could be acquired from the market, and they were exploited without processing. Utilizing the analyzer of Perkin-Elmer 2400C, the analysis of hydrogen, nitrogen and carbon elements was implemented. The FT-IR spectra could be performed utilizing KBr pellets, and it was recorded by employing the spectrometer of Nicolet Impact 750 FTIR with the infrared spectra region from 400 cm^–1^ to 4000 cm^–1^.

### Preparation and characterization for [Zn(H_2_L)_2_(H_2_O)_2_] 1, and [Zn(HL)(2,2'-bpy)] 2

The mixture formed by 30 mg and 0.1 mmol of Zn(NO_3_)_2_·6H_2_O, 0.03 mmol and 9.1 mg of H_3_L and 5 mL of distilled water was sealed into a reactor (20 mL) with Teflon lining, then the product was adjusted to the pH value of 4 with 1 M of HCl and then heated for 72 hours at 120°C. The complex **1ʹ**s flaky colorless crystals were gained after cooling the mixture to environmental temperature, with the yield of 42% according to Zn(NO_3_)_2_·6H_2_O. Anal. Calcd for the C_28_H_20_N_2_O_16_Cu: N, 3.98%; C, 47.77% and H, 2.86%. Found: N 3.83, C 47.55 and H 2.91%. IR (KBr pallet, cm^−1^, Fig S1): 3354(s), 2836(m), 1600(s), 1551(s), 1506(m), 1490(m), 1455(s), 1433(m), 1386(m), 1366(m), 1337(s), 1312(s), 1180(w), 1149(m), 1108(s), 1008(w), 947(w), 907(w), 843(m), 769(m), 737(m), 707(m), 687(m), 613(w), 598(w), 562(w), 491(m).

The mixture synthesized from 30 mg and 0.1 mmol of Zn(NO_3_)_2_·6H_2_O, 0.06 mmol and 18.2 mg of H_3_L, 9.4 mg and 0.06 mmol of 2,2'-bpy and 5 mL of distilled water was sealed into a reactor (20 mL) with Teflon lining, then the product was adjusted to the pH value of 4 with 1 M of HCl and then heated for 72 hours at 120°C. The complex **2ʹ**s colorless massive crystals were gained after cooling the mixture to environmental temperature, with the yield of 58% according to Zn(NO_3_)_2_·6H_2_O. Anal. Calcd for the C_24_H_15_N_3_O_7_Zn: N, 8.04 %; C, 55.14% and H, 2.89 %. Found: N 8.26; C 55.62 and H 2.85%. IR(KBr pallet, cm^−1^): 3423(m), 3063(m), 2934(m), 2783(m), 1593(s), 1563(s), 1489(s), 1451(s), 1380(m), 1364(m), 1338(s), 1312(s), 1233(s), 1162(m), 1150(m), 1108(s), 1026(m), 1005(w), 925(s), 836(m), 776(s), 756(s), 710(m), 689(s), 618(w), 580(w), 535(s).

The data for X-ray can be analyzed through SuperNova diffractometer. For the strength data, it was analyzed via exploiting the CrysAlisPro, and this data was then converted to HKL files. The original structural manners were established by the direct means-based SHELXS, and after that, least-squares method-based SHELXL-2014 was exploited to modify. The anisotropic parameters were mixed with global non-hydrogen atoms. Next, the entire hydrogen atoms were geometrically fixed via applying the AFIX commands to carbon atoms that they adjacent to. Crystal Data for C_28_H_20_N_2_O_16_Zn (**1**, *M* = 705.83 g/mol, CCDC: 2,078,074): orthorhombic, space group Pbca (no. 61), *a* = 16.653(2) Å, *b* = 8.5528(15) Å, *c* = 20.036(3) Å, *V* = 2853.8(7) Å^3^, *Z* = 4, *T* = 296.15 K, μ(MoKα) = 0.947 mm^−1^, *Dcalc* = 1.643 g/cm^3^, 13,700 reflections measured (4.066° ≤ 2Θ ≤ 52.466°), 2817 unique (*R*_int_ = 0.0287, R_sigma_ = 0.0246), which were used in all calculations. The final *R*_1_ was 0.0361 (I > 2σ(I)) and *wR*_2_ was 0.1257 (all data). Crystal Data for C_24_H_15_N_3_O_7_Zn (**2**, *M* = 522.76 g/mol, CCDC: 2,078,075): triclinic, space group P-1 (no. 2), *a* = 8.0632(2) Å, *b* = 11.2635(3) Å, *c* = 13.05920(10) Å, *α* = 72.9630(10)°, *β* = 76.952(2)°, *γ* = 73.741(2)°, *V* = 1075.20(4) Å^3^, *Z* = 2, *T* = 296.15 K, μ(MoKα) = 1.196 mm^−1^, *Dcalc* = 1.615 g/cm^3^, 12,078 reflections measured (5.328° ≤ 2Θ ≤ 52.442°), 4234 unique (*R*_int_ = 0.0407, R_sigma_ = 0.0580) which were used in all calculations. The final *R*_1_ was 0.0596 (I > 2σ(I)) and *wR*_2_ was 0.1531 (all data).

### CHD animal construction

40 D male rats (6–8 weeks, 200–220) used for the CHO animal model construction were provided via the Model Animal Center of Wuhan University (Wuhan, China). This study was approved by the Institutional Animal Care and Use Committee and conducted in accordance with the Institutional and Association for Assessment and Accreditation of Laboratory Animal Care guidelines. All the animal were divided into the control group, model group, compound **1** group and compound **2** group. Then the rats in the model and treatment groups were given isoproterenol hydrochloride solution in order to induce the animal model of CHD. The isoproterenol hydrochloride solution was injected daily subcutaneous. After the treatment, the contents of the TG, TC, HDL, LDL and VLDL were measured for the model evaluation.

### ET and PGI2 ELISA assay

PG12 and ET detection was performed using ELISA Kits (Sigma, US). Supernatants from the plasma were used in the study for determination. This implementation was accomplished fully based on guidance instructions with minor changes. In general, the SD male rats employed in the current study were provided via the Model Animal Center of Wuhan University (Wuhan, China). This study was approved by the Institutional Animal Care and Use Committee and conducted in accordance with the Institutional and Association for Assessment and Accreditation of Laboratory Animal Care guidelines. The isoproterenol hydrochloride solution was daily subcutaneously injected in order to induce the animal model of CHD. Afterwards, the treatment was completed with the two compounds at 5 mg/kg concentration. Subsequently, chloral hydrate was given for anesthesia, and the animal plasma was harvested, and the PGI2 and ET content released by the arterial endothelial cells into plasma was determined.

### Western blotting assay

The real-time RT-PCR assay was conducted in this research to measure the expression levels of GMP140 on the platelet, which reflected the platelet activation levels after compound treatment. Shortly, the isoproterenol hydrochloride solution was daily subcutaneously injected in order to induce the animal model of CHD. Afterwards, the treatment was performed with the two compounds at 5 mg/kg concentration. Subsequently, the platelets could be harvested and the overall RNA in platelets could be extracted via exploiting the Total RNA Extraction kit. The concentration of entire RNA was detected. Afterwards, the relative expression of the GMP140 on the platelet was measured with *gapdh* used as the internal control. This research was repeated at least three times, and the results were presented as mean ± SD.

### Statistical analysis

All the data in this study were presented as means ± standard deviation (SD) from at least three independent experiments and were analyzed with one-way analysis of variance (ANOVA) using GraphPad Prism (GraphPad Software, Inc.). Differences were considered statistically significant at P < 0.05.

## Results and discussion

### Crystal structures

**Figure 1.**
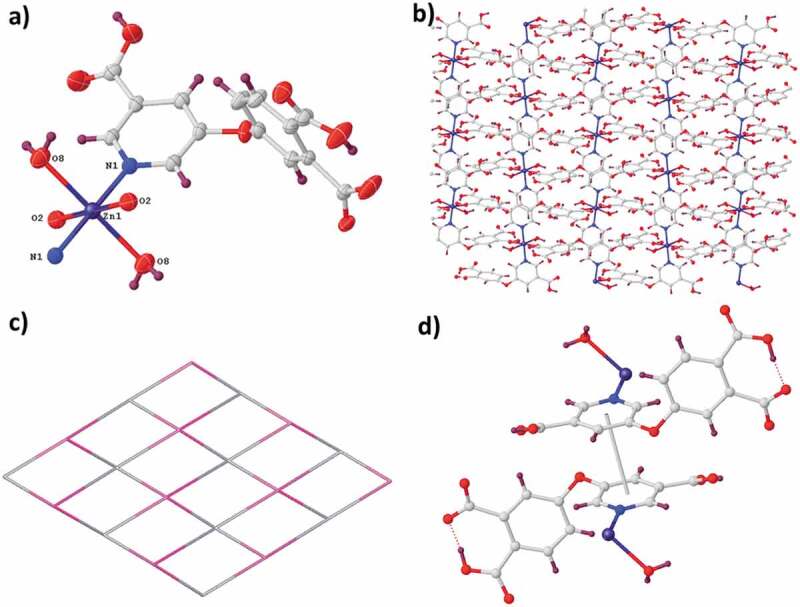
(a) The asymmetry unit for the complex **1**. (b) The **1ʹ**s two-dimensional layered architecture. (c) The four-linked **sql** net of the compound **1**. (d) The interactions of π-π between the neighboring layers.

The complex **1** is part of the space group *Pbca* of orthorhombic system, and its asymmetric unit is constructed from a coordinated molecule of H_2_O, a partly deprotonated ligand of H_2_L^−^ and a separated Zn^2+^ ions (Figure 1(a)). The Zn^2+^ ion exhibited a distorted elongated geometry of octahedron. Two pyridine nitrogen atoms (i.e., N1d and N1) in 2 ligands of H_2_L^−^ and 2 oxygen atoms (namely, O2c and O2b) coming from another two ligands of H_2_L^−^ took over equatorial plane, and two oxygen atoms (i.e., O8d and O8) originating from two diverse coordinated molecules of water took over the axial sites. In the complex **1**, each ligand of H_2_L^−^ connects two centers of Cu^2+^ with 115.88° ether-bond angle. The two aromatic rings are nearly vertical, and the dihedral angle is 87.27 degrees. In the ligand of H_2_L^−^, the two carboxylic group are uncoordinated, and the other one is deprotonated through the monodentate pattern of (κ^1^-κ^0^)-μ_1_-COO-. As reflected in the Figure 1(b), the octahedral ions of Zn^2+^ were connected into two-dimensional layer via the μ_2_-mode molecules of H_2_L^−^. Topologically, each ligand of H_2_L^−^ was applied as the two-linked node, while each center of Zn^2+^ can be considered to be the four-linked node. As a result, the complex **1ʹ**s acquiring architecture is a four-linked uninodal **sql** network with (4^4^ˑ6^2^) point (Schläfli) symbol (Figure 1(c)). Two face-to-face two-dimensional layers are linked through the interactions of π-π to the general complex **1ʹ**s ultimate three-dimensional supramolecular net (Figure 1(d)).

**Figure 2.**
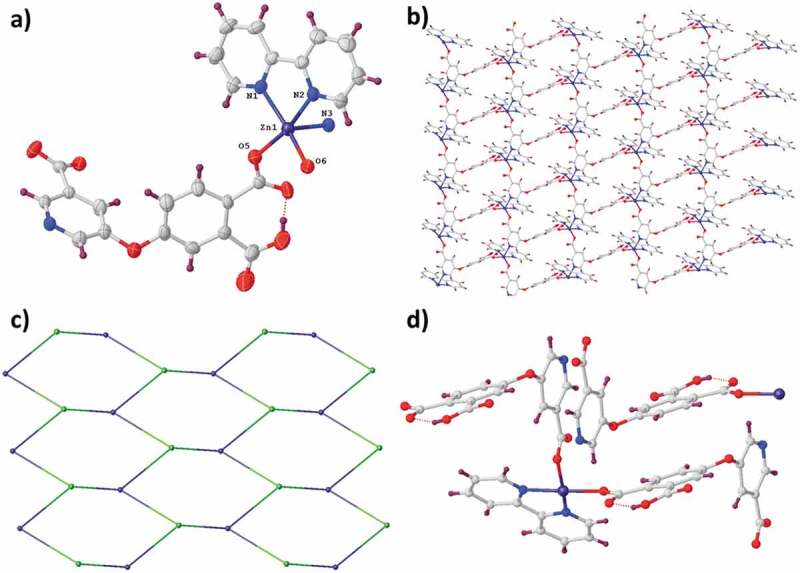
(a) The asymmetry unit for the compound **2**. (b) The **2ʹ**s two-dimensional net. (c) The three-linked topology for the compound **2**. (d) The interactions of π-π between the consecutive layers.

The compound **2** was crystallized in the space group *P-1* of triclinic system, and in its asymmetric unit, there exist a molecule of 2,2'-bpy, a ligand of HL^−^ as well as a Zn^2+^ ion, as reflected in Figure 2(a). The Zn^2+^ exhibited an elongated and distorted coordination geometry of octahedron, and in equatorial sites, it was occupied via two carboxylic acid oxygen atoms originating from two ligands of HL^2−^ and two pyridine nitrogen atoms in a molecule of 2,2'-bpy, while in the axial position existed another carboxylic acid oxygen atom and a nitrogen atom. In compound **2**, each ligand of HL^2−^ connects three centers of Zn^2+^, and between two aromatic rings, the dihedral angle is 77.05° and the ether-bond angle is 117.36°. In the ligand of HL^2−^, the carboxyl groups are deprotonated partly, where the deprotonated carboxylic acids employs two diverse types of coordination patterns, namely, (κ^1^-κ^0^)-μ_1_-COO^−^ and (κ^1^-κ^1^)-μ_1_-COO^−^. As illustrated in Figure 2(b), μ_3_-coordinated molecules of HL^2−^ connect the centers of Zn^2+^ into two-dimensional layer architecture on plane *ab*. It should be noted that the **2ʹ**s two-dimensional layer is very different from the **1ʹ**s two-dimensional layer. In topology, both the Zn^2+^ center and ligand of HL^2−^ can be considered as a three-linked node, thus the **2ʹ**s intricate structure is classified to a three-linked uninodal **hcb** topology with (6^3^) point (Schläfli) symbol (Figure 2(c)). From the two-dimensional layer, the molecules of 2,2'-bpy are protruded, which are regularly distributed on the same layer side. It should be noted that the molecules of 2,2'-bpy possess no actual contribution to the generation of **2ʹ**s two-dimensional layer architecture, they only saturate the Zn^2+^ center coordination. With the interactions of π … π between the ligands of HL^2−^, these two-dimensional supramolecular bilayers in depth self-assemble into the **2ʹ**s three-dimensional supramolecular net, as reflected in Figure 2(d).

**Figure 3.**
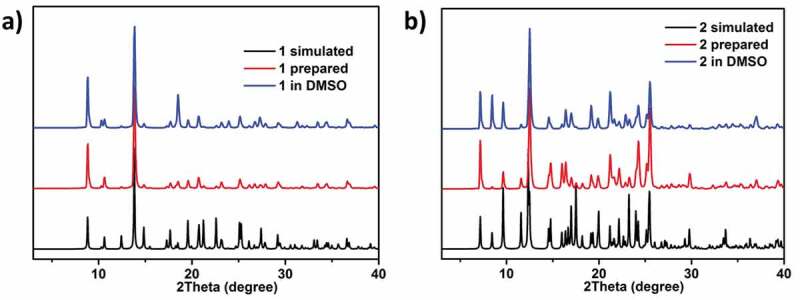
The PXRD patterns for complex **1** (a) and **2** (b).

In order to determine these complexes regarding the phase purity, the experiments of powder X-ray diffraction (PXRD) were carried out. The simulated and experimental PXRD peak positions are consistent, which shows that the crystal structures are the genuine representative for the bulk crystal (Figure 3). The strength difference may be because of the crystal samples’ preferred orientation. Considering the following bioactivity tests, it is necessary to study the framework stability of complex **1** in the injection solvent DMSO. Because complex **1** could not be dissolved in the organic solvents and water, we used its stock solution in the following bioactivity tests. With this in mind, about 100 mg of the as-prepared crystalline samples of **1** were taken in a mortar. It was then ground manually for 30 min by using a pestle. The produced fine powders were soaked in 20 mL DMSO and subjected to the ultrasonic treatments for 2 hours to obtain the well dispersed solution. After standing for 2 days, the fine powders could be recovered via centrifugation, and their PXRD measurements showed that the PXRD profiles of the treated samples show good match with those of the as-prepared samples, reflecting their good stability in the above conditions.

### Compound significantly reduced the inflammatory response in arterial endothelial cells

**Figure 4.**
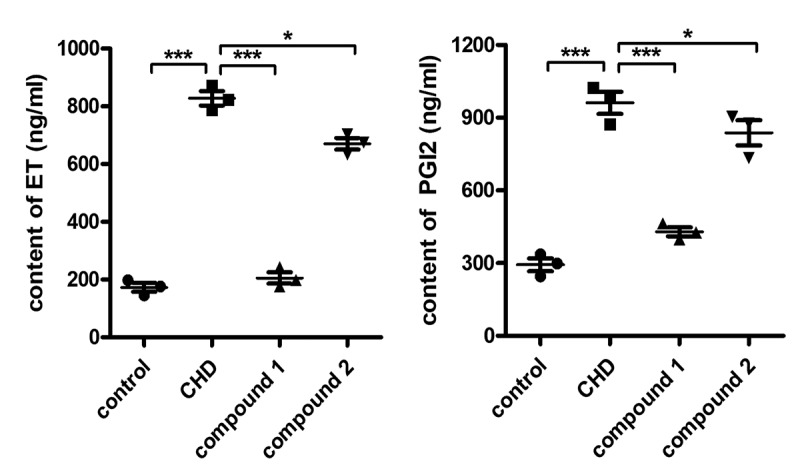
Significantly reduced inflammatory response in arterial endothelial cells after compound treatment. The animal model of CHD was established and then the treatment was completed with compound at 5 mg/kg concentration. The content of the ET and PGI2 released by the arterial endothelial cells into the plasma was explored via exploiting the ELISA detection kit. * means P < 0.05 and *** means P < 0.005.

For the sake of assessing the compounds’ protective activity on CHD, the compounds’ protective effect against the arterial endothelial cells was first determined. Thus, in the current investigation, ELISA detection was accomplished for the determination of the PGI2 and ET content released by the arterial endothelial cells into plasma. As the outcomes illustrated in the Figure 4, we can observe that contrast to control group, the model group has a much higher PGI2 and ET content, with P value < 0.005. After treated via compound, the **1** showed significantly inhibitory effect on ET and PGI2 releasing, but the **2** exhibited only minor effect against the releasing of PGI2 and ET.

### Compound obviously inhibited the expression levels of GMP140 on the platelet

**Figure 5.**
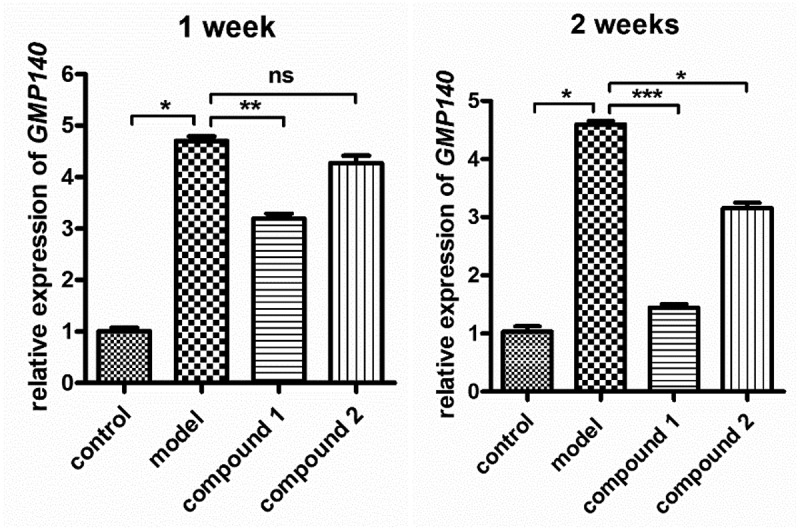
Obviously inhibited GMP140 expression levels on the platelet after compound treatment. The animal model of CHD was established and then the treatment was completed with compound at 5 mg/kg concentration. Real-time RT-PCR assay was accomplished and the expression levels of GMP140 for the platelet were detected.

As described above, the compound showed excellent inhibitory influence against the PGI2 and ET content released via the arterial endothelial cells into plasma. During the CHD procession, the arterial endothelial cells functional dis-regulation could lead to platelet activation. Thus, in this present research, the platelet activation state was further evaluated after compound treatment. The information in Figure 5 reveals that the expression levels of GMP140 on the platelet was much higher in the model group in contrast to the control group. With the **1ʹ**s treatment, the GMP140 expression levels on the platelet was decreased, but the **2** possesses no influence against the expression levels of GMP140.

## Conclusion

To recap briefly, we have created two fresh transition metal coordination polymers in success through the reaction of 5-(3,4-dicarboxylphenoxy) nicotic acid (H_3_L), the semi-rigid multifunctional tricarboxylate, with the reaction conditions of solvothermal. The analysis of CHN and the architectures of the two complexes were investigated. The analysis for the diffraction of single-crystal X-ray suggests that the **1** reveals the separated two-dimensional layer architecture having a four-linked uninodal topology (4^4^ˑ6^2^), and this layer self-assembles into an ultimate three-dimensional supramolecular net. For the **2**, the ancillary nitrogen-donor 2.2'-bpy ligand was only employed as the decoration; hence, the compound **2** also reveals a two-dimensional layer architecture. The ELISA assay results indicated that the **1** was more outstanding than the complex **2** on the reduction of PGI2 and ET content released by the arterial endothelial cells into plasma. Additionally, the expression levels of GMP140 on the platelet were also inhibited by compound **1**, which is stronger than compound **2**. In the end, the **1** was more superior to the complex **2** to be a candidate for the CHD treatment by reducing inflammatory response in arterial endothelial cells and platelet activation state.

## Data Availability

IR spectra of **1** and **2** (Fig. S1). The information could be found in the supporting information file.
